# NAC Family Transcription Factors in Tobacco and Their Potential Role in Regulating Leaf Senescence

**DOI:** 10.3389/fpls.2018.01900

**Published:** 2018-12-21

**Authors:** Wei Li, Xiaoxu Li, Jiangtao Chao, Zenglin Zhang, Weifeng Wang, Yongfeng Guo

**Affiliations:** Tobacco Research Institute, Chinese Academy of Agricultural Sciences, Qingdao, China

**Keywords:** NAC family, leaf senescence, *NtNAC080*, bioinformatic analysis, transcriptome, tobacco

## Abstract

The NAC family is one of the largest families of plant-specific transcription factors (TFs) and NAC proteins play important regulatory roles in a variety of developmental and stress response processes in plants. Members of the NAC family TFs have been shown to be important regulators of leaf senescence in a number of plant species. Here we report the identification of the NAC family in tobacco (*Nicotiana tabacum*) and characterization of the potential role of some of the tobacco NAC TFs in regulating leaf senescence. A total of 154 NAC genes (*NtNACs*) were identified and clustered together with the Arabidopsis NAC family into fifteen groups (a-o). Transcriptome data analysis followed by qRT-PCR validation showed that the majority of the senescence-up-regulated *NtNAC*s fall into subgroups NAC-b and f. A number of known senescence regulators from Arabidopsis also belong to these two subgroups. Among these senescence-up-regulated *NtNACs*, *NtNAC080*, a close homolog of AtNAP, is a positive regulator of leaf senescence. Overexpression of *NtNAC080* caused early senescence in Arabidopsis leaves and *NtNAC080* mutation induced by Cas9/gRNA in tobacco led to delayed leaf senescence.

## Introduction

Senescence is the final stage of leaf development and is critical for plants’ life cycle. As an important mechanism of evolutionary fitness, nutrients are remobilized from senescing leaves to other parts of the plant such as developing seeds and young leaves through the senescence process. Leaf senescence is an active, genetically controlled and highly regulated process accompanied by differential expression of 1000s of genes, including those with regulatory functions (Lohman et al., 1994; Hajouj et al., 2000; van der Graaff et al., 2006; Lim et al., 2007; Balazadeh et al., 2008; Guo, 2013; Ali et al., 2018). At the transcriptional level, the drastic changes in gene expression during leaf senescence are driven by transcription factors (TFs), the activities of which are triggered by a combination of aging signals and environmental factors (Guo, 2013). Previous studies have identified a number of TFs that participated in the process of leaf senescence from a number of protein families such as NAC, WRKY, and MYB proteins. These TF proteins act as switches to cause differential gene expression by binding to specific cis-acting elements of target gene promoters, resulting in the activation and/or suppression of target genes during senescence (Gregersen and Holm, 2007; Balazadeh et al., 2008; Besseau et al., 2012; Zhang and Gan, 2012; Shah et al., 2013).

The plant-specific NAC TFs compose one of the largest plant TF families. A NAC protein possesses a well-conserved N-terminal NAC domain (∼160 amino acids) and a variable C-terminal transcription regulatory region (TRR) (Ooka et al., 2003; Olsen et al., 2005b). The DNA binding ability of NAC TFs is confined to the NAC domain which can be divided into five subdomains (A-E). The highly conserved Subdomains C and D may be responsible for DNA binding, whereas Subdomain A may be involved in dimerization. The divergent Subdomains B and E may be responsible for functional diversity of the NAC proteins. The highly divergent C-terminal TRR region of NAC TFs is suggested to confer the regulation diversity of transcriptional activation activities (Ernst et al., 2004; Olsen et al., 2005a; Balazadeh et al., 2011; Kjaersgaard et al., 2011; Christiansen and Gregersen, 2014; Lindemose et al., 2014). Besides, an α-helical transmembrane (TM) motif present in some NAC proteins has a function to anchor onto either endoplasmic reticulum or plasma membranes (Seo et al., 2008), which may play important regulatory roles in abiotic stress responses (Sang-Gyu et al., 2008; Li et al., 2016).

The NAC family proteins have been shown to participate in diverse biological processes, including development of root and shoot apical meristems (Takada et al., 2001; Vroemen et al., 2003), organogenesis (Yamaguchi et al., 2010), hormone signaling (Xie et al., 2002; Kim et al., 2006; Jensen et al., 2008), fruit ripening (Shan et al., 2012; Ríos et al., 2017; Tranbarger et al., 2017), response to biotic and abiotic stresses (Nakashima et al., 2007, 2012; Huang et al., 2015; Yan et al., 2017), fiber, and secondary cell wall development (Mitsuda et al., 2007; Mitsuda and Ohme-Takagi, 2008; Zhong et al., 2008; Li et al., 2012; Chai et al., 2015; Zhang et al., 2018). In addition, NAC proteins have been shown to play important roles in regulating leaf senescence in various plant species (Lee et al., 2012; Fan et al., 2015; Podzimska-Sroka et al., 2015; Takasaki et al., 2015; Pimenta et al., 2016; Mao et al., 2017). A total of twenty NAC genes were present in an ESTs library of Arabidopsis senescent leaf (Guo et al., 2004). In a gene expression profiling study, about 30 NAC genes showed altered expression during Arabidopsis leaf senescence (Breeze et al., 2011). Among the senescence-up-regulated NAC TF genes, AtNAP was first identified as a positive regulator of leaf senescence. Overexpression of *AtNAP* led to premature senescence, whereas leaf senescence of the *atnap* knockout mutant plants was significantly delayed (Guo and Gan, 2006). In addition to AtNAP, NAC TFs ORS1, ORE1, and ATAF1 have been shown to play a role in promoting senescence in Arabidopsis as positive regulators while JUB1 and VNI2 are negative regulators of senescence (Balazadeh et al., 2011; Yang et al., 2011; Wu et al., 2012; Rauf et al., 2013; Garapati et al., 2015).

The roles of NAC TFs in regulating senescence seem to be conserved across species. AtNAP homologous genes have so far been reported in a variety of plant species including rice, maize, wheat, soybean, kidney bean, peach, tomato, petunia, potato, poplar, tall fescue, and bamboo (Guo and Gan, 2014). Like AtNAP, the homologs of rice, kidney bean, bamboo, and cotton have been shown to be expressed in senescing leaves but not in non-senescing ones and, more importantly, the homologs function as AtNAP orthologs because they complemented the Arabidopsis *nap* null mutants (Guo and Gan, 2006; Chen et al., 2011; Fan et al., 2015). Furthermore, the AtNAP ortholog in rice, OsNAP, was shown to have the same regulatory role in rice leaf senescence (Liang et al., 2014). RNA silencing of the maize homolog, ZmNAP, caused a stay green phenotype in maize (Zhang et al., 2012). Knocking down of the NAP ortholog in cotton, GhNAP, also caused a significant delay in leaf senescence (Fan et al., 2015).

The NAC family has been characterized in a number of plant species. These studies indicated that there are 105 NAC genes in Arabidopsis (Ooka et al., 2003), 151 in rice (Nuruzzaman et al., 2010), 163 in poplar (Hu et al., 2010), 74 in grape (Wang et al., 2013), 152 in soybean (Le et al., 2011), and 97 in *Medicago truncatula* (Ling et al., 2017). Using gene-space sequence reads (GSR) from methylation filtrated tobacco genomic DNA libraries, an earlier study on tobacco TFs reported 152 NAC domain genes in tobacco (*Nicotiana tabacum* L.) (Rushton et al., 2008). Taking advantage of the recently available high quality genome sequences of tobacco and its two ancestor species *(N. sylvestris* and *N. tomentosiformis*) (Gerstel, 1960; Sierro et al., 2013), in the current study we have identified the complete set of NAC proteins in tobacco, and have analyzed their gene structures, protein motifs and phylogeny. We also did a comprehensive analysis of the expression profile of all *NtNAC* genes using RNA-Seq data as well as quantitative real-time PCR validation. Moreover, potential roles of tobacco NAC members in regulating leaf senescence were predicted based on expression pattern and sequence homology, and function of one of the senescence-up-regulated NAC genes, *NtNAC080*, in leaf senescence was investigated.

## Materials and Methods

### Database Search and Sequence Retrieval of NAC Proteins From *Nicotiana tabacum*

The tobacco genomic sequences were downloaded from SGN (Sol Genomics Network)^1^. The Hidden Markov Model (HMM) profile of NAC domain (PF02365) retrieved from Pfam^2^ was used to conduct HMM search against the annotated protein database with an *E*-value cutoff of 1.0 using HMMER (v3.0) (Johnson et al., 2010). All non-redundant hits within expected values were collected and each newly identified hit was subsequently used as a query to perform BLASTP search against the annotated tobacco genome. The protein sequences obtained from the two above-described approaches were combined and redundant entries were removed manually. The resulted non-redundant sequences were manually analyzed using InterPro to ensure presence of the NAC domain (Apweiler et al., 2001). Moreover, TMHMM Server ver.2.0 was used to predict the membrane-bound *NtNAC* members (Krogh et al., 2001).

### Gene Structure and Motif Analysis

Exon-intron structures of tobacco *NAC* genes were analyzed and illustrated with the Gene Structure Display Server (GSDS)^3^ by comparison of gene CDS sequences with genomic DNA sequences obtained from the Sol Genomics Network. The program MEME version 4.3.0 was employed for the detection of conserved motifs in tobacco NAC proteins^4^. MEME was run locally with the following parameters: distribution of motif occurrences, zero or one per sequence; maximum number of motifs, 10; optimum motif width, ≥6 and ≤200 (Bailey et al., 2015).

### Multiple Sequence Alignment and Phylogenetic Analysis

Multiple sequence alignments of full-length tobacco NAC amino acid sequences were performed using the MAFFT program with the default parameters together with 105 Arabidopsis NAC protein sequences and two representative NAC proteins (StNAC010 and StNAC039) from potato (*Solanum tuberosum* L.) which have been reported to be in the Solanaceae-specific subfamily (TNACs) of the NAC family (Katoh and Standley, 2013). Sequences of Arabidopsis and potato NAC proteins were downloaded from TAIR10^5^ and SGN^6^, respectively. Un-rooted phylogenetic trees were constructed via MEGA 6.06 using the Neighbor-Joining (NJ) method with the following parameters: Poisson model and bootstrap values of 1000 replicates. Pairwise deletion mode was employed to make sure that the divergent C-terminal domains could contribute to the topology of the phylogenetic trees (Tamura et al., 2013).

### Plant Materials and Growth Conditions

Common tobacco (*Nicotiana tabacum* L. Cv. K326) plants were grown in the field. Middle leaves (Leaf No. 9-10 from the base of a tobacco plant) were collected at 15, 45, 65, 75 days after topping (DAT) (defined as YL, young leaf; ES, early senescing leaf; MS, mid-senescing leaf; LS, late senescing leaf, respectively) for analyzing gene expression during leaf senescence. All harvested leaves were wrapped in aluminum foil, immediately placed in liquid nitrogen and stored at −80°C until used. Three biological replicates were analyzed for each time point. In gene functional analysis, The T1 plants of *NtNAC080* CRISPR-Cas9 transgenic lines and wild-type plants (K326) were grown in the greenhouse under normal growth conditions.

Arabidopsis Col-0 and transgenic plants were grown in a plant growth chamber (Conviron, Canada) at 22°C with a relative humidity of 55% under long-day conditions (16 h light/8 h dark) with white light illumination (120 μmol photons/m^2^s).

### Gene Expression Profiling

The data used for expression profiling of tobacco *NAC* genes were from the tobacco Illumina RNA-seq data generated in the Guo lab (Li et al., 2017) (archived by NCBI under the accession numbers: SRP102153). For RNA-Seq data of tobacco leaf senescence, RPKM values of the 154 *NtNAC* genes were retrieved and normalized. A heatmap was generated based on the log_2_ fold-change values at 25/35/45/55/65/75DAT when compared with 15DAT and visualized with R package (R Development Core Team, 2013). In addition, the data used for expression profiling of Arabidopsis *NAC* genes (*ANAC* genes) were retrieved from AtGenExpress leaf developmental data bySchmid et al. (2005). Based on the previous Guo et al. analysis, we performed differential expression analysis of all the *ANAC* genes between the fully expanded leaves (young leaves) and senescing leaves (Guo and Gan, 2012). Genes that are up-regulated twofold or more were designated as being senescence up-regulated *ANACs*.

### RNA Isolation and Quantitative Real-Time RT-PCR (qRT-PCR) Analysis

Total RNA was extracted according to the method previously described (Li and Guo, 2018). Genomic DNA was eliminated from the RNA samples via DNase I (Takara bio inc., Otsu, Japan) treatment for 30 min at 37°C. The quality and concentrations of RNA samples were determined using a Nanodrop 2000 spectrophotometer (Thermo Fisher Scientific, Wilmington, DE, United States). First-strand cDNAs were synthesized using the RevertAid H Minus First Strand cDNA Synthesis Kit (Thermo Scientific) with Oligo(dT) primers. qRT-PCR was performed on an ABI7500 Real-Time PCR System (Applied Biosystems, Foster City, CA, United States) with the reaction mixtures comprising 1 μL template cDNA, 1 μL each of forward and reverse primers (0.3 μM each) and 10 μL FastStart Universal SYBR Green Master (Rox, Roche Applied Science). For tobacco, *NtActin* was used as an internal control. *NtCP1* (SAG12 homolog in tobacco) and *NtRBCS* were used as senescence markers. For Arabidopsis, *AtActin* and *AtSAG12* were used as the internal control and senescence marker, respectively. The relevant primers are given in Supplementary Table S1. All reactions were run in triplicate. The relative gene expression values were analyzed using the 2^−Δ Δ Ct^ method.

### Overexpressing *NtNAC080* in Arabidopsis

The coding sequence of *NtNAC080* was PCR amplified from K326 cDNA using the PrimeSTAR HS DNA polymerase (Takara Bio, Japan) and cloned into the binary vector *pCHF3* (a modified *pPZP212* vector) at the *Bam*HI/*Sac*I sites after the CaMV 35S promoter (Hajdukiewicz et al., 1994). Primers used in vector construction are listed in Supplementary Table S2. The construct was then used to transform Arabidopsis using the Agrobacterium floral dip method (Clough and Bent, 1998). Transgenic Arabidopsis plants were selected on kanamycin (50 mg/L) plates. Presence of the transgene was PCR confirmed using genomic DNA from the leaves of putative transformants. Abundance of the *NtNAC080* transcript was estimated via qRT-PCR using cDNA from senescing leaves of the transgenic plants.

### CRISPR-CAS9-Mediated Mutation Induction in Tobacco

The DNA sequences of *NtNAC080*, *NtNAC028*, *NtNAC083*, and *NtNAC110* are highly similar. To produce a gRNA specific to *NtNAC080*, an online software^7^ was used for synthesizing two DNA oligos, *NtNAC080*-gRNA1 and *NtNAC080*-gRNA2, The *NtNAC080*-sgRNA forward and reverse primers were denatured by heating at 95°C for 3 min and annealed to form a double-stranded DNA. Thereafter, the double-stranded DNA was inserted into the *pORE*-Cas9/gRNA vector at the *Bsa*I site (Gao et al., 2015). Tobacco cultivar K326 was transformed with the resulting construct via Agrobacterium-mediated transformation as described earlier (Horsch et al., 1989). The shoots of putative transformants were selected in Murashige and Skoog (MS) medium containing 0.1 mg/L 1-naphthylacetic acid (NAA), 1 mg/L 6-dimethylaminopurine (6-BA), 50 mg/L kanamycin (Kan), and 500 mg/L cefotaxime sodium (Cef). Putative Kan-resistant transformants were transferred to soil pots and grown under greenhouse conditions. The genomic DNA of transgenic tobacco plants from Kan selection were extracted using the DNeasy Plant Mini Kit (Qiagen, CA, United States). The DNA fragments containing the Cas9/gRNA target sequences were amplified by PCR using PrimeSTAR HS DNA polymerase (Takara Bio, Japan). After purification, the PCR product was cloned to the *pEASY*-Blunt Zero vector (Transgene, China) and the DNA from the single colonies were sequenced to detect the types of mutation. For the target gene edited tobacco plants, selfing was performed and the homozygous mutants were acquired from T1 transgenic plants. The relevant primers are given in Supplementary Table S2.

### Chlorophyll Extraction and Quantification

Chlorophyll was extracted and quantified as described previously (He and Gan, 2002). Briefly, 10 mg of freeze-dried leaf tissue was extracted with 1 mL of 95% ethanol in the dark for 24 h with agitation. The supernatant was quantified via spectrophotometric measurement at 649 and 665 nm. Three biological replicates were used for each sample.

## Results

### Identification and Phylogenetic Analysis of NAC Family Members in Tobacco

To identify NAC genes in tobacco, Hidden Markov Model (HMM) search was performed against the Sol Genomics Network database^8^ using the Pfam NAC domain (PF02365) as query. Newly identified hits were used as queries to carry out BLASTP search until no further hit can be obtained. After manual removal of redundant hits, all the putative NAC proteins were further screened using the Pfam^9^ and the Interpro^10^ programs to ensure that each protein has a N-terminal NAC domain. Following this procedure, a total of 154 non-redundant NAC family proteins were identified in tobacco. The tobacco *NAC* genes were named *NtNACs* followed by Arabic numbers. The length of the NtNAC proteins ranges from 130 to 645 amino acids (aa) with an average of 330 aa. The detailed information of NAC family genes in tobacco, including accession numbers, protein lengths, protein sequences, conserved domains and similarities to their Arabidopsis orthologs etc., is listed in Supplementary Tables S3, S4.

**FIGURE 1:**
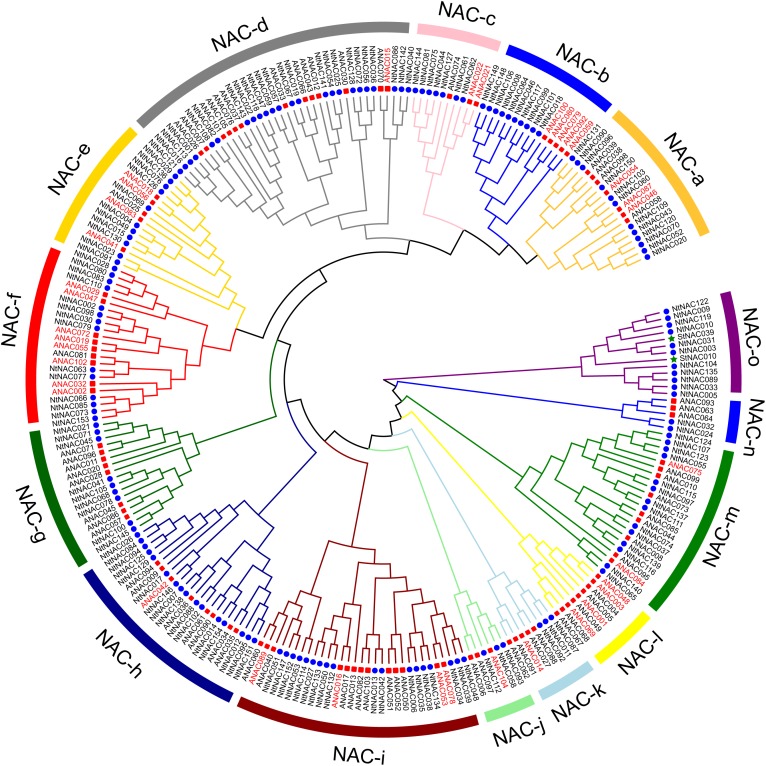
Phylogenetic tree of NAC proteins of tobacco, Arabidopsis and potato. Multiple sequence alignments of NAC proteins were performed by MAFFT program with default parameters. The un-rooted phylogenetic tree was constructed by MEGA 6.06 with the Neighbor-Joining (NJ) methods using the following parameters: Poisson model and bootstrap values (1000 replicates). Pairwise deletion mode was employed to make sure that the divergent C-terminal domains could contribute to the topology of the phylogenetic tree. The tree was divided into 15 phylogenetic subgroups, designated as NAC-a to NAC-o. Members of tobacco, Arabidopsis and potato were denoted by blue spots, red squares and green asterisks. *ANAC* genes which were specifically up-regulated in senescing leaves are indicated by red color. Ratios of expression change for individual *ANAC* genes were presented in Supplementary Table S5.

To study the evolutionary relationship between the tobacco NAC proteins and known NACs from other plant species, an unrooted phylogenetic tree was constructed from alignments of 154 NtNACs, 105 Arabidopsis ANACs and 2 representative potato NAC protein sequences (Figure 1). All the NAC proteins were divided into 15 distinct subgroups: NAC-a to NAC-o. The tobacco NAC members demonstrated an interspersed distribution in all the subgroups except the subgroup NAC-l, in which all members are from Arabidopsis. The subgroup NAC-o, on the other hand, contains no NAC from Arabidopsis. Further alignment analysis suggested that proteins in subgroup NAC-o shared sequence characteristics of TNACs, which have been reported to be the Solanaceae-specific NAC subfamily (Rushton et al., 2008). The two representative TNAC potato proteins, StNAC010 and StNAC039 (Singh et al., 2013), were also grouped in this subgroup, suggesting NAC-o being a Solanaceae-specific NAC subgroup.

### Gene Structure and Protein Motif Analysis of the NAC Gene Family in Tobacco

**FIGURE 2:**
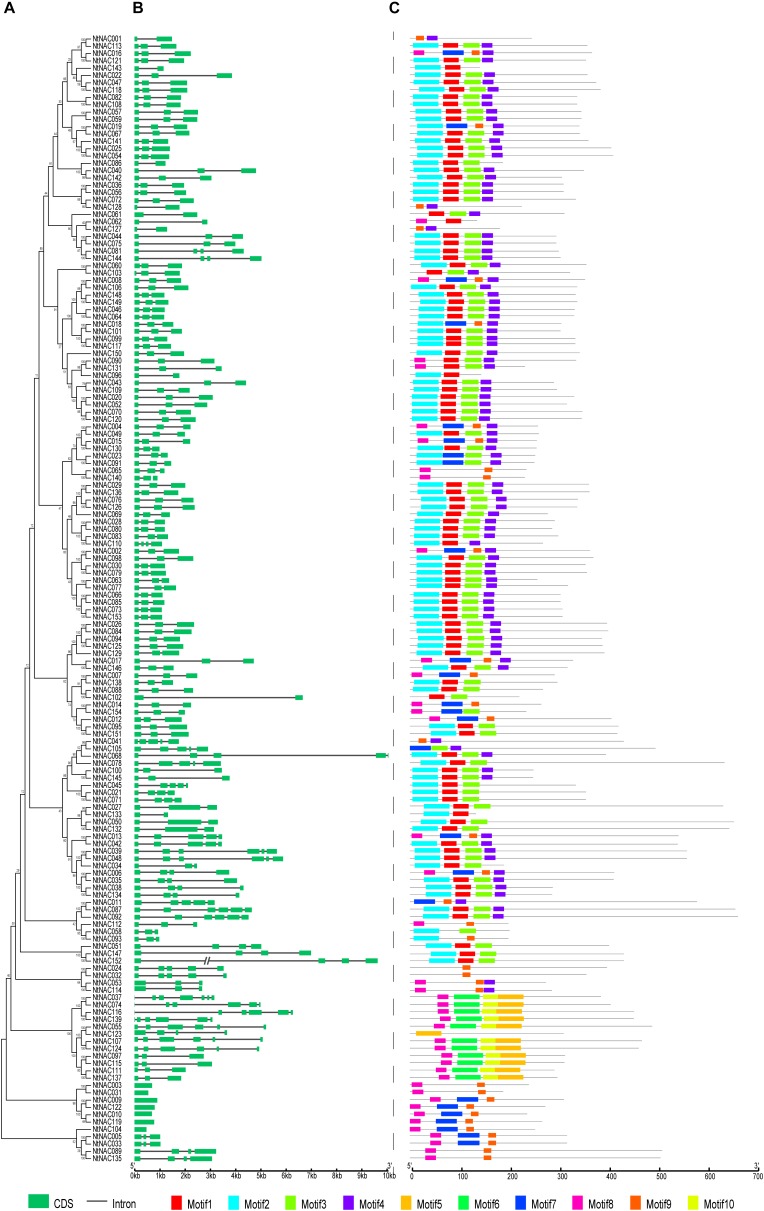
Gene structures and motif clades of 154 NtNAC transcription factors. **(A)** Multiple alignments of 154 *NtNAC* genes were executed by Clustal X 1.83 and the phylogenetic tree was constructed using MEGA 6.06 by the Neighbor-Joining (NJ) method with 1,000 bootstrap replicates. **(B)** Exons and introns were represented by green boxes and black lines, respectively. Scale represents the sizes of exons and introns can be estimated using the scale at the bottom. **(C)** The MEME motifs were shown as different-colored boxes at the N-terminal indicating the NAC domain region.

To gain insights into the structural diversity of the *NtNAC* genes, the exon/intron structure of the coding sequences of individual *NtNACs* in tobacco was analyzed (Figure 2B). In general, members of the same subgroup share similar exon/intron structure and gene length. For example, the *NAC* genes in subgroups NAC-a, NAC-b, NAC-d, NAC-e, and NAC-h contain one or two introns. The NAC-f members have two introns except *NtNAC110*, which harbors three introns. In addition, 7 of the *NAC* genes have no intron, all of which belong to subgroup NAC-o. The members in subgroups NAC-i and NAC-m on the other hand, have more variable gene structures. Among the 154 *NtNACs*, the shortest *NtNAC* gene is 474 bp (*NtNAC104*) long, whereas *NtNAC068* is the longest *NtNAC* gene with a 10 kb genomic sequence.

Using the MEME program, 10 conserved motifs, named Motif 1-10, were identified in the NtNAC proteins, all located within the N-terminal region which are highly conserved for DNA-binding, as described previously (Singh et al., 2013; Wei et al., 2016; Figure 2C and Supplementary Figure S1). Motif 2/8, Motif 1/7, Motif 3, and Motif 4 comprise the NAC DNA-binding domain while other motifs are dispersed around the NAC domain. Similar motif organization was observed within the same subgroup or in closely related subgroups (Figures 1, 2A,C). For instance, only NAC members in the Solanaceae-specific subgroup NAC-o contain both Motifs 8 and 9. Subgroup NAC-f mainly contains Motifs 1, 2, 3, and 4. Most members of the subgroup NAC-m comprise Motif 3, 5, 8, and 10. A conserved nuclear localization signal (NLS) was identified within Motif 3 (Supplementary Figure S1). As expected, the C-terminal transcriptional regulation region (TRR) is variable.

A trans-membrane (TM) region with a predicted-helix was identified in the far C-terminal region of 13 NtNACs proteins, which belonging to subgroups NAC-i, NAC-k and NAC-o. Remarkably, subgroup NAC-i contains most of membrane-bound NAC members of tobacco and Arabidopsis, including 8 NtNACs and 7 ANACs (Supplementary Table S3). There are 18 membrane-associated NAC TFs in Arabidopsis, 5 in rice, 11 in soybean and 14 in potato (Kim et al., 2007, 2010; Singh et al., 2013). A number of Arabidopsis membrane-bound NAC proteins have been shown to be involved in biotic and abiotic stress responses (Kim et al., 2007, 2010; Lee et al., 2012).

### Prediction of *NtNAC* Genes Involved in Leaf Senescence Using Transcriptome Data

NAC TFs play an important regulatory role in leaf senescence (Li and Guo, 2014). Transcriptomic studies have shown that approximately one third of the NAC genes were up-regulated during leaf senescence in Arabidopsis, highlighting their importance in senescence regulation (Breeze et al., 2011). Using the publicly available gene-chip based data by Schmid et al. (2005), 36 *ANAC* genes which were up-regulated twofold or more during leaf senescence were identified (Supplementary Table S5). Interestingly, 13 of the senescence-up-regulated *ANACs* belong to the phylogenetic subgroups NAC-b and NAC-f, which totally contain 14 ANAC genes from Arabidopsis. Moreover, several ANAC TFs that have been previously identified as positive regulators of leaf senescence, including ANAC029/AtNAP, ANAC059/ORS1, ANAC092/ORE1/AtNAC2, ANAC002/ATAF1 (Guo and Gan, 2006; Balazadeh et al., 2011; Rauf et al., 2013; Garapati et al., 2015) were clustered together in the NAC-b and NAC-f subgroups. *NAC* genes with same functions showed a tendency to fall into the same subgroup (Fang et al., 2008; Hu et al., 2015; Wei et al., 2016). We thus predict that the *NAC* genes of NAC-b and NAC-f subgroups might play an important role in regulating leaf senescence.

**FIGURE 3:**
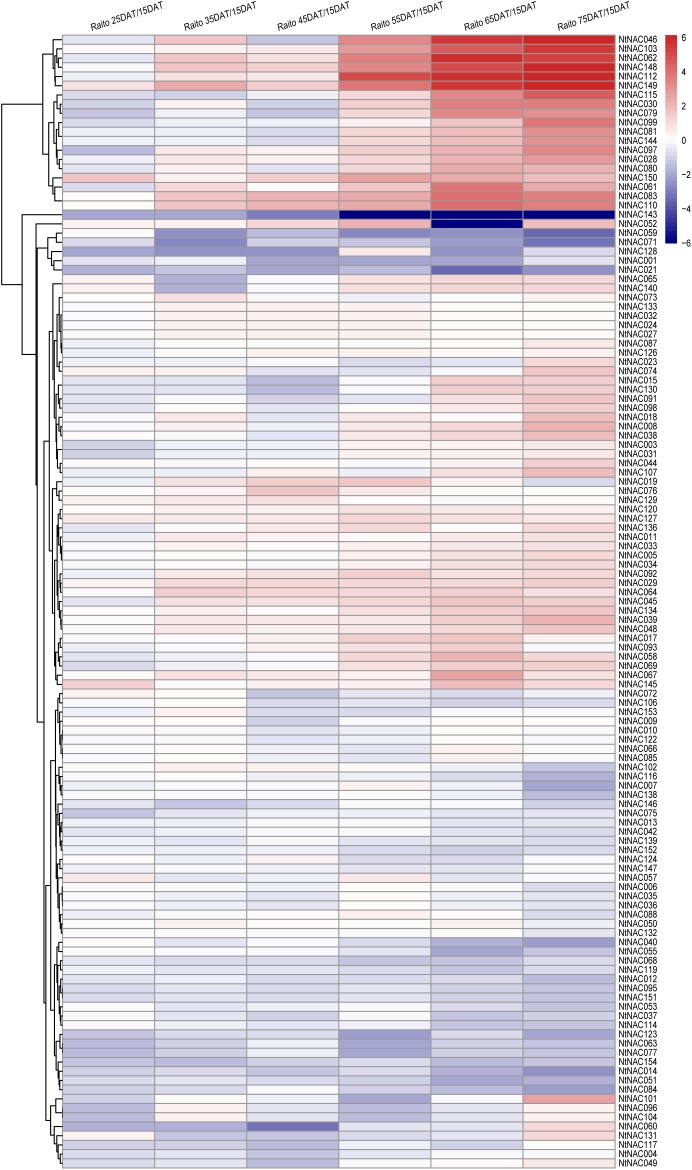
Heatmap representation and hierarchical clustering of *NtNAC* genes at different stages of leaf senescence. The Illumina RNA-Seq data was reanalyzed, and the heatmap was generated based on the log2 fold-change values at 25/35/45/55/65/75DAT when compared with 15DAT. Changes in expression levels were displayed from blue (down-regulated) to red (up-regulated), as shown in the color gradient at the top right corner. DAT, days after topping.

To explore the patterns of tobacco *NAC* expression during leaf senescence, we performed a comprehensive analysis of *NtNAC* genes expression profiles at different stages of leaf senescence based on data retrieved from our earlier RNA-Seq study of tobacco leaf senescence (Li et al., 2017). We were able to obtain transcripts data from most of the *NtNACs* (147 out of 154) from the dataset (Supplementary Table S6). As previously reported in Arabidopsis and other species (Breeze et al., 2011; Christiansen and Gregersen, 2014), the analysis of expression changes indicated significant transcriptional responses of the *NtNAC* genes during tobacco leaf senescence. Twenty four of the *NtNACs* were up-regulated fourfold or more at least at one time point from 25DAT to 75DAT compared with 15DAT (Figure 3 and Supplementary Table S6). Interestingly, 11 of these 24 genes were clustered in subgroups NAC-b and NAC-f, and are closely related to the Arabidopsis senescence-regulating NAC genes, including AtNAP/ANAC029, ANAC002, ANAC059, and ANAC092 (Figure 1). In addition, four other *NtNAC* genes in subgroups NAC-b and NAC-f, including *NtNAC008*, *NtNAC018*, *NtNAC064*, and *NtNAC098*, showed an up-regulation of 2–4-fold and most of the remaining genes in the two subgroups showed somewhat increased expression during senescence. *NtNACs* in subgroups NAC-b and NAC-f shared similar up-regulation patterns during senescence as their homologs in Arabidopsis, suggesting that these two subgroups may play significant roles in regulating leaf senescence and these regulatory roles might be conserved between species.

### Quantitative PCR Analysis of Selected *NtNAC* Genes During Tobacco Leaf Senescence

**FIGURE 4:**
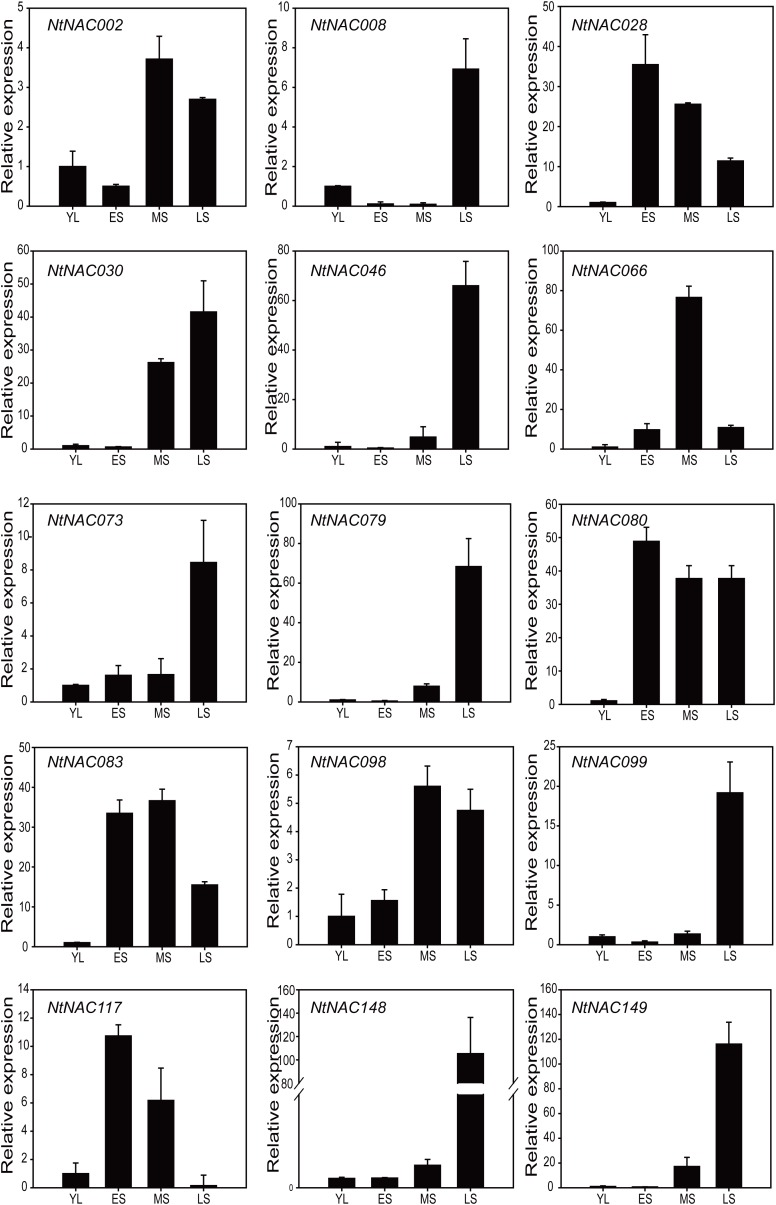
Expression profiles of 15 representative *NtNAC* genes analyzed by qRT-PCR during tobacco leaf senescence. The relative expression ratio of each gene was calculated relative to its expression at the first stage (YL). qRT-PCR data were normalized using tobacco *Actin* gene. The bars are standard deviations (SD) of three biological replicates.

To validate the result of RNA-Seq analysis, we analyzed the expression patterns of 15 selected *NtNAC* genes from the subgroups NAC-b and NAC-f during leaf senescence using quantitative RT-PCR (qRT-PCR). Data from 4 leaf developmental stages, including fully expanded, non-senescent leaf (YL), early senescent leaf (ES), middle senescent leaf (MS), and late senescent leaf (LS) were used (Supplementary Figure S2) and all of the 15 *NtNAC* genes tested were found to be up-regulated during senescence, with a fold change of two or more (Figure 4). Among them, *NtNACs* including *NtNAC002*, *NtNAC030*, *NtNAC046*, *NtNAC079*, *NtNAC098*, and *NtNAC149* were up-regulated at the MS stage with high level expression maintained until the LS stage. Expression of some other *NtNACs* genes (*NtNAC008*, *NtNAC073*, *NtNAC099*, and *NtNAC148*) were induced only at the LS stage. Expression of *NtNAC028*, *NtNAC080*, *NtNAC083*, and *NtNAC117* increased rapidly at the ES stage, suggesting potential roles of these genes in regulating the onset of leaf senescence. Phylogenetic analysis showed that *NtNAC028*, *NtNAC080*, and *NtNAC083* were closely related to *ANAC029/AtNAP*, which acts as a key positive regulator of leaf senescence (Guo and Gan, 2006). Similarly, *NtNAC117* was in the same clade that contained the Arabidopsis senescence-associated genes *ANAC059/ORS1* and *ANAC092/ORE1*, which also play significant roles in regulating senescence (Balazadeh et al., 2010, 2011; Rauf et al., 2013). We therefore predicted that these four genes (*NtNAC028*, *NtNAC080*, *NtNAC083*, and *NtNAC117*) might function as key regulators of leaf senescence in tobacco.

### Overexpression of *NtNAC080* Induces Early Leaf Senescence in Arabidopsis

**FIGURE 5:**
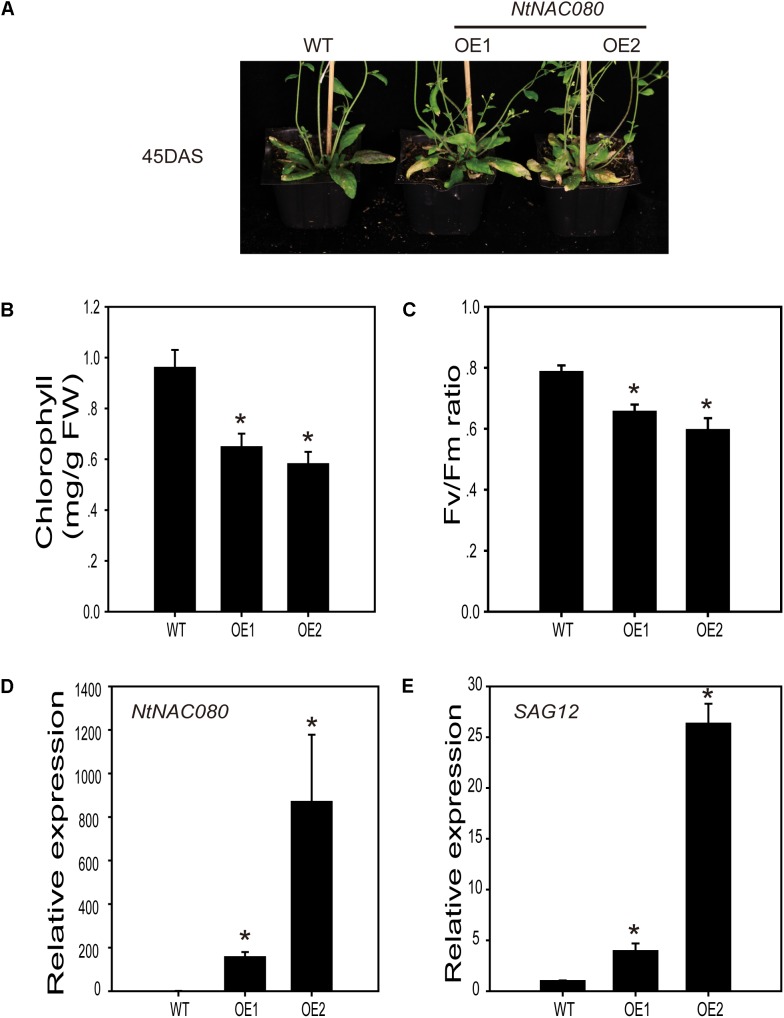
Overexpression of *NtNAC080* in Arabidopsis plants causes precocious senescence. **(A)** Phenotypes of wild-type (WT) and transgenic plants (OE1 and OE2). The picture was taken at 45 days after sowing. **(B,C)** Chlorophyll content and Fv/Fm ratios in leaf 5 of age-matched WT and two OE lines (leaf number counted from the bottom of the plant). **(D,E)** qRT-PCR analysis of expression of *NtNAC080* and *SAG12* in WT and two OE lines. *AtActin* was used as the internal control. The bars are standard deviations (SD) of three biological replicates. ^∗^Significant difference using student’s *t*-test (*p* < 0.05).

To further assess the function of senescence-associated *NtNAC* genes *in planta*, we obtained transgenic Arabidopsis plants overexpressing *NtNAC080*, expression of which increased significantly at early stages of leaf senescence in tobacco, with a 48-folds change at the ES stage. Two lines of *NtNAC080*-overexpressing transgenic Arabidopsis, with expression of *NtNAC080* confirmed by qRT-PCR (Figure 5D), exhibited a premature senescence at 45 days after sowing (DAS) (Figure 5A). The precocious leaf yellowing phenotype was also supported by changes in total chlorophyll content and the maximal photochemical efficiency of PSII (Fv/Fm), which reflects the photochemical quantum efficiency of PSII and photosynthetic activity (Figures 5B,C). Expression of the senescence-specific cysteine protease *SAG12*, which is a widely used molecular marker of leaf senescence, was strongly induced in leaves of the two *NtNAC080*-overexpressing lines (Figure 5E). These data indicated that, like its close homolog ANAC029/AtNAP in the NAC-f subgroup, NtNAC080 also acts as a positive regulator of leaf senescence.

### CRISPR-Mediated *NtNAC080* Mutations Delay Leaf Senescence in Tobacco

**FIGURE 6:**
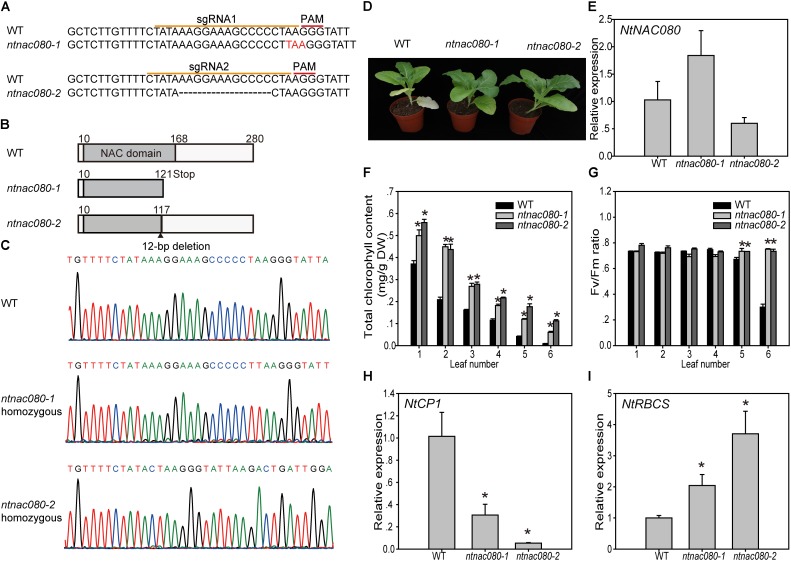
Delayed leaf senescence phenotype of the CRISPR-mediated *ntnac080* mutant compared with that of age-matched wild-type plants. (**A–C)** The construction of NtNAC080 CRISPR-Cas9 lines. In **(A,C)** sequence results of mutants were aligned to the reference genome sequence. In **(B)** putative mutated NtNAC080 proteins in homozygous plants (T1). Gray zones were NAC conserved domains (10–168 aa). In *ntnac080-1* plants, 1 bp insertion led to a stop after position P121. In *ntnac080-2* plants, a 12 bp deletion caused a deletion of four amino acids and transition of one amino acid at position 117. **(D)** Phenotypes of *ntnac080* mutants. **(E)** qRT-PCR analysis of expression of *NtNAC080* in *ntnac080-1* mutants. **(F,G)** Chlorophyll content and Fv/Fm ratios in leaf 1–6 of age-matched wild-type (K326) and mutant lines (leaf number counted from the top of plant). **(H,I)** qRT-PCR analysis of expression of *NtCP1* and *NtRBCS* genes in leaf 5 of wild-type (K326) and mutant lines. *NtActin* was used as the internal control. The bars are standard deviations (SD) of three biological replicates. ^∗^Significant difference using student’s *t*-test (*p* < 0.05).

To confirm the function of *NtNAC080* in regulating tobacco leaf senescence, we mutated *NtNAC080* using the CRISPR/Cas9 system in tobacco. Two gRNAs (sgRNA1 and sgRNA2) targeting the NAC domain of *NtNAC080* were created and used in Agrobacterium-mediated transformation of tobacco plants (Figure 6A). Twelve T0 plants for *NtNAC080* sgRNA1 and 14 T0 plants for *NtNAC080* sgRNA2 were obtained after kanamycin screening. PCR-amplified target regions of these plants were sequenced to identify mutations. Eleven of the 12 (91.7%) sgRNA1 and 9 of the 14 (64.3%) sgRNA2 T0 plants were found to contain mutations with deletion or insertion of nucleotides (Supplementary Table S7). Homozygous mutant plants were obtained from the T1 generation and two representative lines *ntnac080-1* and *ntnac080-2*, were further used for phenotype analysis (Figure 6C). The *ntnac080-1* line had a 1 bp insertion at the 3′ end of sgRNA1 sequence which introduced a stop codon after position P121, and the *ntnac080-2* plants harbor a 12 bp deletion causing a deletion of four amino acids and transition of one amino acid at position 117 (Figure 6B). In addition, we have also sequenced *NtNAC028*, the closest homolog of *NtNAC080*, in the two *ntnac080* mutants. The sequencing results confirmed that these CRISPR mutants contain no mutation on *NtNAC028* gene. Thus these mutant lines might express abnormal NtNAC080 proteins with a truncated/mutated NAC domain (Figures 6B,C). Both *ntnac080-1* and *ntnac080-2* plants showed a similar delayed senescence phenotype compared to WT (K326) tobacco (Figure 6D). Quantitative RT-PCR indicated no difference in transcript levels of *NtNAC080* between these two mutants and WT plants (Figure 6E). We also assessed the progression of leaf senescence between mutants and WT by measuring changes in chlorophyll content and the maximal photochemical efficiency of PSII (Fv/Fm). Under glasshouse conditions, chlorophyll levels in individual leaves (leaf 1-6, numbered from the top to the bottom of a plant) of the two mutants were generally higher than that in counterpart leaves of the age-matched WT plants (Figure 6F). The Fv/Fm ratios in leaf 5 and 6 of the mutants plants were also higher than that in counterpart leaves of WT plants (Figure 6G). Furthermore, we determined the expression of senescence-related marker genes *NtCP1* (SAG12 homolog in tobacco) and *NtRBCS* in leaf 5. As shown in Figures 6H,I, the expression of *NtCP1* was lower and *NtRBCS* was higher in two mutants. These data indicated that NtNAC080 acts as a positive regulator of leaf senescence in tobacco.

## Discussion

As one of the most important transcription factor families in plant, the *NAC* gene family plays a pivotal role in regulating various development and physiological processes. Although the functions of a number of *NAC* genes have been investigated in Arabidopsis and model crops (Guo and Gan, 2006; Garapati et al., 2015; Pimenta et al., 2016; Mao et al., 2017), little is known about this gene family in the economic crop *Nicotiana tabacum*. An earlier study has identified 152 NAC domain genes from methylation filtrated tobacco genomic DNA libraries but detailed information of this gene family was not available (Rushton et al., 2008). The biological functions of most tobacco *NAC* genes remain unknown except two *NAC* genes that have been functionally characterized (Fu et al., 2013; Han et al., 2014). In this study we identified 154 NAC proteins in the tobacco genome and compared them with the Arabidopsis NAC proteins. In many cases two or more tobacco NAC proteins were found to be highly homologous to an Arabidopsis NAC, most likely due to the whole-genome duplication during tobacco tetraploidization. The duplicated genes may have evolved to have diversified functions.

Based on phylogenic analysis, the NAC proteins from tobacco and Arabidopsis were divided into 15 distinct subgroups. The exon/intron structure and motif compositions of *NACs* were highly conserved in each subgroup. Earlier studies suggested that NAC genes from the same subgroup tend to have similar functions (Fang et al., 2008; Hu et al., 2015; Wei et al., 2016). For instance, subgroup NAC-i contains most of membrane-bound NAC members of tobacco and Arabidopsis, suggesting a potential role of the subgroup NAC-i members in regulating stress responses.

Two other subgroups of the NAC family proteins, namely subgroup NAC-b and f, seem to have an important role in regulating leaf senescence. A great numbers of the senescence up-regulated *ANACs* are clustered in subgroups NAC-b and NAC-f, and a number of previously characterized senescence-regulating ANAC TFs are also in these subgroups (Figure 1). We examined the expression of 15 *NtNAC* genes in subgroups NAC-b and NAC-f via qRT-PCR analysis and all of these *NtNAC* genes showed a significant up-regulation during tobacco leaf senescence (Figure 4).

The role of NAC family TFs in leaf senescence seems to be conserved between different plant species. In Arabidopsis, ∼30 out of 105 *NAC* genes are up-regulated during leaf senescence, including *AtNAP*, *ORE1*, and *ORS1* (Breeze et al., 2011). In barley, ∼15 out of 47 studied *NAC* genes are up-regulated during senescence (Christiansen and Gregersen, 2014). Phylogenetic analysis of the barley NAC proteins suggested that 12 of these 15 up-regulated *NAC* genes belonged to two subfamilies, which also harbor a number of previously characterized senescence-regulating NAC TFs from other species, such as Arabidopsis, rice, and wheat. Based on sequence homology and expression profiles, several putative regulators of senescence in barley, including *HvNAC005*, *HvNAC027*, and *HvNAC029/HvNAM1*, have been identified (Christiansen et al., 2011; Podzimska-Sroka et al., 2015). Interestingly, majority of the *ANAC* genes in these two above-mentioned barley NAC subfamilies also belong to NAC-b and f in the phylogenetic tree in this study (Figure 1).

A number of studies have shown that *NAC* genes are induced in senescent leaves and that overexpression of some *NAC* genes alters senescence process in plants. In Arabidopsis, overexpression of either *AtNAP* or *ORE1* promoted leaf senescence, while overexpression of *JUB1* or *VNI2* led to delayed senescence (Guo and Gan, 2006; Yang et al., 2011; Wu et al., 2012; Rauf et al., 2013). In rice, overexpression of *OsNAC2* and *OsNAP* resulted in early senescence, while overexpression of *OsNAC106* resulted in delayed senescence (Liang et al., 2014; Sakuraba et al., 2015; Mao et al., 2017). Meanwhile, a wheat NAC transcription factor, *NAM-B1*, accelerates senescence and increases nutrient remobilization from leaves to developing grain in wheat (Uauy et al., 2006). It is noteworthy that the genes which promote the process of leaf senescence, such as *AtNAP*, *OsNAP*, *NAM-B1, ORE1*, and *OsNAC2*, were phylogenetically clustered together (Podzimska-Sroka et al., 2015), indicating that the *NAC* genes with similar biological functions are closely related.

However, the genes that function in delaying senescence were found randomly distributed on the phylogenetic tree. For example, VNI2 was a member of the subgroup NAC-e while JUB1 belonged to the NAC-h subgroup (Figure 1). Many of the NAC genes in the NAC-e subgroup were up-regulated while genes of the NAC-h subgroup were down-regulated in senescent leaves (Supplementary Tables S5, S6). Four NtNACs genes, including NtNAC004, NtNAC049, NtNAC015, and NtNAC130 were closely related to the negative senescence regulator VNI2. Similarly, NtNAC146 was clustered in the same clade with JUB1. At the transcriptional level, *NtNAC049*, *NtNAC015*, and *NtNAC130* were down-regulated at the early stage of senescence (from 15DAT to 55DAT) then up-regulated after 65DAT. Whereas, *NtNAC004* and *NtNAC146* were continuously down-regulated during leaf senescence. In Arabidopsis, *JUB1* and *VNI2* showed significantly up-regulation during senescence. The difference in expression patterns of these NAC genes between tobacco and Arabidopsis suggests that in comparison with their Arabidopsis homologs, the negative senescence regulators in the tobacco NAC family might have different mechanisms in controlling leaf senescence. The senescence-inhibiting NAC genes may have more complex evolutionary relationships compared to the senescence-promoting NACs.

In the current study, to explore the function of *NtNACs* in regulating leaf senescence, we have identified orthologous pairs between *NtNACs* and *ANACs* based on sequence similarity. Due to the polyploid genome of common tobacco, we have generally identified two or more *NtNACs* highly homologous to each Arabidopsis *NAC* gene. For instance, two homologous gene pairs (*NtNAC028*/*NtNAC080* and *NtNAC083*/*NtNAC110*) were found to be orthologous to the key senescence- regulating *AtNAP*. The tobacco NAP homologs exhibited similar patterns of transcript accumulation during leaf senescence (Figure 3). Furthermore, *NtNAC028*, *NtNAC080*, and *NtNAC083* were induced rapidly at early senescence (ES) stage in the qRT-PCR analysis, indicating that these genes may perform similar function as *AtNAP* in regulating the onset of leaf senescence. To validate this hypothesis, we carried out functional analysis of some of these genes. Our results showed that overexpression of *NtNAC080* induced Arabidopsis early leaf senescence, whereas *ntnac080* mutants obtained via CRISPR-Cas9 strategy delayed leaf senescence of tobacco leaves. Similar results were obtained for *NtNAC028* overexpressing and knocking-out studies in our laboratory (unpublished data). The deduced proteins of *NtNAC028* and *NtNAC080* are closely related to each other (91.87% sequence identity). Overexpression of both *NtNAC028* and *NtNAC080* induced senescence while mutation in either gene caused delay in leaf senescence (Figures 5, 6, unpublished data), suggesting that as a result of gene family expansion in tobacco, these homologous *NtNAP* genes share similar but not completely redundant roles in regulating leaf senescence. This may lead to more plasticity in senescence regulation. Like *NtNAC028*/*NtNAC080*, expression the other homologous pair (*NtNAC083/ NtNAC110*) was also found increased during leaf senescence, but the transcripts of these two genes showed an obvious increase at 35DAT, before the initiation of leaf senescence (Figure 3). The difference in expression patterns suggested that *NtNAC083* and *NtNAC110* may play distinct roles in regulating leaf senescence and other development processes in tobacco. Further investigation is needed in elucidating the molecular mechanisms underlying the regulation of leaf senescence by *NtNAP* genes as well as other NAC family genes in tobacco.

Overall, this study demonstrates that a number of *NAC* genes might be involved in regulating leaf senescence in tobacco and have revealed a potential structure-function relationship between NAC family members as putative senescence regulators.

## Author Contributions

YG conceived the project. YG and WL designed the research. WL performed plant transformation, the phenotypic analysis, and qRT-PCR verification. XL performed NtNAC TF identification, phylogenetic analysis, gene structure, and motif analysis. WL, XL, and JC performed transcriptomic analyses. ZZ and WW designed CRISPR-Cas9 experiment and generated the constructs. WL, YG, and XL wrote the manuscript.

## Conflict of Interest Statement

The authors declare that the research was conducted in the absence of any commercial or financial relationships that could be construed as a potential conflict of interest.

## Footnotes

1 http://solgenomics.net/

2 http://pfam.sanger.ac.uk/

3 http://gsds.cbi.pku.edu.cn/

4 http://meme.sdsc.edu

5 http://www.arabidopsis.org/

6 http://solgenomics.net/

7 http://www.multicrispr.net/index.html

8 http://solgenomics.net/

9 http://pfam.xfam.org/

10 http://www.ebi.ac.uk/interpro/search/sequence-search

## Supplementary Material

FIGURE S1 Sequence logos for conserved motifs identified in NtNACs by MEME analysis.

FIGURE S2 Senescence stages of tobacco leaves used in this study. YL, young leaf; ES, an early senescent leaf; MS, a mid-senescent leaf; LS, a late senescent leaf.

TABLE S1 Sequences of primer pairs used for qRT-PCR analysis.

TABLE S2 Sequences of primer pairs used for vector construction and transgenic plants sequencing.

TABLE S3 Characteristics of *NAC* gene family in tobacco.

TABLE S4 Protein sequences of *NAC* gene family in tobacco.

TABLE S5 Expression change ratio of *ANAC* genes between senescent leaves and non-senescing leaves.

TABLE S6 Expression Patterns of 154 *NtNACs* during tobacco leaf senescence.

TABLE S7 Summary of targeted mutagenesis at the *NtNAC080* locus in transgenic plants (T0).
