# Supplementary material for: NAC Family Transcription Factors in Tobacco and Their Potential Role in Regulating Leaf Senescence
Source: Front Plant Sci. 2018 Dec 21;9:1900. doi: 10.3389/fpls.2018.01900 (PMC6308388; doi:10.3389/fpls.2018.01900)
Supplement: FIGURE S1 — Sequence logos for conserved motifs identified in NtNACs by MEME analysis. [file Data_Sheet_1.pdf]

Supplementary Information

NAC family transcription factors in tobacco and their potential role in regulating leaf senescence

Wei Li[1], Xiaoxu Li[1], Jiangtao Chao [1], Zenglin Zhang [1], Weifeng Wang [1] and Yongfeng Guo[1,*]

[1] Tobacco Research Institute, Chinese Academy of Agricultural Sciences, Qingdao, Shandong 266101, China

[*]Corresponding author: Yongfeng Guo, Email: guoyongfeng@caas.cn, Tel: +86-532-66715256

Number of supplementary figures: 2

Number of supplementary tables: 7

Figure S1

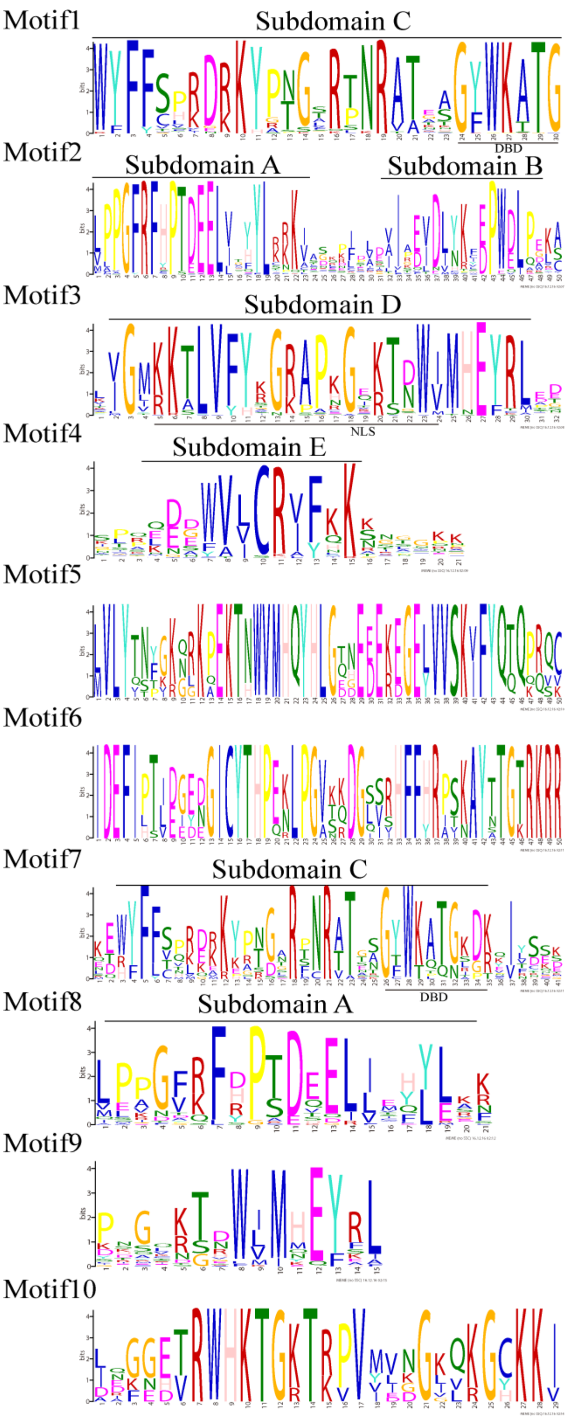

Figure S1 Sequence logos for conserved motifs identified in NtNACs by MEME analysis.

Figure S2

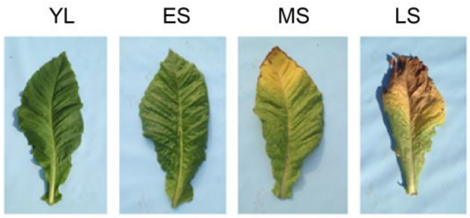

Figure S2 Senescence stages of tobacco leaves used in this study. YL, young leaf; ES, an early senescent leaf; MS, a mid-senescent leaf; LS, a late senescent leaf.
