# Supplementary material for: NAC Family Transcription Factors in Tobacco and Their Potential Role in Regulating Leaf Senescence
Source: Front Plant Sci. 2018 Dec 21;9:1900. doi: 10.3389/fpls.2018.01900 (PMC6308388; doi:10.3389/fpls.2018.01900)
Supplement: TABLE S1 — Sequences of primer pairs used for qRT-PCR analysis. [file Table_1.docx]

**Supplementary Table S1. Sequences of primer pairs used for qRT-PCR analysis.**

| Name | Forward primer | Reverse primer |
| --- | --- | --- |
| *NtNAC002* | 5’-CTGGATGAAGAAGTTGAAAGCAGAATC-3’ | 5’-TACTGCCTGAACCCCAGATTTAATG-3’ |
| *NtNAC008* | 5’-TAACCAAATAGTCCCAGTTCAGAGC-3’ | 5’-GTAACTCCAAAGGCAATCAAGACTC-3’ |
| *NtNAC028* | 5’-ATTGCCAAGGGCTTGTTCACTATCT-3’ | 5’-GTGTTTTGGTTGTTAGCGTTGTATG-3’ |
| *NtNAC030* | 5’-GTACCGGAAATCAATCTCAGGTGCA-3’ | 5’-ACCGAGTTCGTGTAGGCTTGTAACC-3’ |
| *NtNAC046* | 5’-AGAGATAGTAAGTTTATCACAAGAAACAGTT-3’ | 5’-CAAAGATAGTCAAGATACTGTGGTCC-3’ |
| *NtNAC066* | 5’-TACAATAAGAAGGGTACAATGGAGAAGAC-3’ | 5’-AGATCGAAATCGGCAACGGCAGAAT-3’ |
| *NtNAC073* | 5’-CTGGTTATTGGAAAGCGACTGGTGC-3’ | 5’-GCATAAAACCCAATCATCCAGCCT-3’ |
| *NtNAC079* | 5’-CGGGAAATCAATCTCAGGTGC-3’ | 5’-TCTCTATTTCTTCGTCCACTCCACC-3’ |
| *NtNAC080* | 5’-TTCACGACTATTTGATGACAATGCTAAC-3’ | 5’-TATTGGTCATTGTGTTTTGGTTGTT-3’ |
| *NtNAC083* | 5’-GAGAAATCTCAGCTAGGGGAAGTGT-3’ | 5’-CTCATAGTACCCCATACGACAGTTCT-3’ |
| *NtNAC098* | 5’-GGATGAAGAAGTTGAAAGCGGGATT-3’ | 5’-AGATTTAATGTCTGGTTAGGACACTGAAT-3’ |
| *NtNAC099* | 5’-CATCCAACAGATGAAGAGCTTATCACAT-3’ | 5’-GTTGCTCTGTTTGTCCTCTGCCCTGT-3’ |
| *NtNAC117* | 5’-CCAACAGATGAAGAGCTTATCACTC-3’ | 5’-TGCTCTGTTTGTCCTCTGTCCTGT-3’ |
| *NtNAC148* | 5’-CAGCAGAGTTTGAAAAAGGAGAAAGAG-3’ | 5’-ATCCTGTTGAACGACAGATGATGTAAT-3’ |
| *NtNAC149* | 5’-GAACCAAACAGTCCCTTTTCCTCAA-3’ | 5’-CTACTGAAGCTCTACTCATCTCTCTGC-3’ |
| *NtActin* | 5’-ACCTCTATGGCAACATTGTGCTCAG-3’ | 5’-CTGGGAGCCAAAGCGGTGATT-3’ |
| *NtCP1* | 5’-CAGTGGCTAATCAACCTGTTTCGG-3’ | 5’-ACACCACTTGAATAGAACTGGAAATCG-3’ |
| *NtRBCS* | 5’-CGAAACTCTCTCATACCTTCCCGA-3’ | 5’-CATGGTCCAGTATCTGCCGTCATA-3’ |
| *AtActin* | 5’-TGTGCCAATCTACGAGGGTTT-3’ | 5’-TTTCCCGCTCTGCTGTTGT-3’ |
| *AtSAG12* | 5’-TCCAATTCTATTCGTCTGGTGTGT-3’ | 5’-CCACTTTCTCCCCATTTTGTTC-3’ |
