# Supplementary material for: NAC Family Transcription Factors in Tobacco and Their Potential Role in Regulating Leaf Senescence
Source: Front Plant Sci. 2018 Dec 21;9:1900. doi: 10.3389/fpls.2018.01900 (PMC6308388; doi:10.3389/fpls.2018.01900)
Supplement: TABLE S2 — Sequences of primer pairs used for vector construction and transgenic plants sequencing. [file Table_2.docx]

**Supplementary Table S2. Sequences of primer pairs used for vector construction and transgenic plants sequencing.**

| Name | Forward primer | Reverse primer |
| --- | --- | --- |
| pCHF3-*NtNAC080*  (Isolation of gene) | 5’-GAGCTCCCCTTCTATTTATTTCCTTGACC-3’ | 5’-GGATCCTCACTGGTATTGAAAGGCTGGA-3’ |
| *NtNAC080*-sgRNA1 | 5’-GATTGTATAAAGGAAAGCCCCCTAA-3’ | 5’-AAACTTAGGGGGCTTTCCTTTATAC-3’ |
| *NtNAC080*-sgRNA2 | 5’-GATTGCTATAAAGGAAAGCCCCCTA-3’ | 5’-AAACTAGGGGGCTTTCCTTTATAGC-3’ |
| pORE-*NtNAC080*  (sequencing primer) | 5’-ATCCCCGAAGTTGATGTCTA-3’ | 5’-AAGTCTTACCCTCATGGAGCC-3’ |
