# Supplementary material for: NAC Family Transcription Factors in Tobacco and Their Potential Role in Regulating Leaf Senescence
Source: Front Plant Sci. 2018 Dec 21;9:1900. doi: 10.3389/fpls.2018.01900 (PMC6308388; doi:10.3389/fpls.2018.01900)
Supplement: TABLE S7 — Summary of targeted mutagenesis at the NtNAC080 locus in transgenic plants (T0). [file Table_7.docx]

**Supplementary Table S7 Summary of targeted mutagenesis at the NtNAC080 locus in transgenic plants (T0).**

|  | Plant lines | Sequence |  |
| --- | --- | --- | --- |
|  | WT | GCTCTTGTTTTCTATAAAGGAAAGCCCCCTAAGGGTATT |  |
| ­­  sgRNA1 | #1  #10 | GCTCTTGTTTTCTATAAAGGAAAGCCCCCCTAAGGGTATT  GCTCTTGTTTTCTATAAAGGAAAGCCCCCTTAAGGGTATT | +1  +1 |
|  | #2  #3  #4  #5  #7 | GCTCTTGTTTTCTATAAAGG-------------------TAAGGGTATT  GCTCTTGTTTTCTATAAAGGAAAG------CCTAAGGGTATT  GCTCTTGTTTTCTATAAAGGAAAG----CCCTAAGGGTATT  GCTCTTGTTTTCTATAAAGGAAAG--CCCCTAAGGGTATT  GCTCTTGTTTTCTATAAAGGAAAGCCCCCTTAAGGGTATT | -9  -3  -2  -1  +1 |
|  | #6 | GCTCTTGTTTTCTATAAAGG-------------------TAAGGGTATT  GCTCTTGTTTTCTATAAAGGAAAG------CCTAAGGGTATT  GCTCTTGTTTTCTATAAAGGAAAG----CCCTAAGGGTATT  GCTCTTGTTTTCTATAAAGGAAAG--CCCCTAAGGGTATT  GCTCTTGTTTTCTATAAAGGAAAGCCCCCTTAAGGGTATT  GCTCTTGTTTTCTATAAAGGAAAGCCCCCCTAAGGGTATT | -9  -3  -2  -1  +1  +1 |
|  | #8 | GCTCTTGTTTTCTATAAAGGAAAGCCCCCTTAAGGGTATT | +1 |
|  | #9  #11 | GCTCTTGTTTTCTATAAAGG-------------------TAAGGGTATT  GCTCTTGTTTTCTATAAAGGAAAG------CCTAAGGGTATT  GCTCTTGTTTTCTATAAAGGAAAG----CCCTAAGGGTATT  GCTCTTGTTTTCTATAAAGGAAAG--CCCCTAAGGGTATT | -9  -3  -2  -1 |
| sgRNA2 | #12 | GCTCTTGTTTTCTATA------------------------- CTAAGGGTATT  GCTCTTGTTTTCTATAAAGGAAAGCCCCCCTAAGGGTATT | -12  +1 |
|  | #13  #19 | GCTCTTGTTTTCTATAAAGGAAAG - CCCCTAAGGGTATT | -1 |
|  | #14 | GCTCTTGTTTTCTATAAAGGAAAGCCCCCCTAAGGGTATT | +1 |
|  | #15 | GCTCTTGTTTTCTATAAAGGAAAGCCCCCCTAAGGGTATT  GCTCTTGTTTTCTATAAAGGAAAGCCCCCATAAGGGTATT | +1  +1 |
|  | #16 | GCTCTTGTTTTCTATA------------------------- CTAAGGGTATT | -12 |
|  | #17 | GCTCTTGTTTTCTATA------------------------- CTAAGGGTATT  GCTCTTGTTTTCTATAAAGGAAAGCCCCCCTAAGGGTATT | -12  +1 |
|  | #18 | GCTCTTGTTTTCTATAAAGGAAAGCCCCCCTAAGGGTATT  GCTCTTGTTTTCTATAAAGGAA------------CCTAAGGGTATT | +1  -5 |
|  | #20 | GCTCTTGTTTTCTATA------------------------- CTAAGGGTATT  GCTCTTGTTTTCTATAAAGGAAAGCCCCCCTAAGGGTATT  GCTCTTGTTTTCTATAAAGGAAAG - CCCCTAAGGGTATT | -12  +1  -1 |

The target sequences of sgRNA1 and sgRNA2 partially overlapped.
